# Time- and angle-resolved photoemission spectroscopy (TR-ARPES) of TMDC monolayers and bilayers

**DOI:** 10.1039/d2sc04124c

**Published:** 2022-12-06

**Authors:** Fang Liu

**Affiliations:** a Department of Chemistry and the PULSE Institute, Stanford University Stanford California 94305 USA fliu10@stanford.edu

## Abstract

Many unique properties in two-dimensional (2D) materials and their heterostructures rely on charge excitation, scattering, transfer, and relaxation dynamics across different points in the momentum space. Understanding these dynamics is crucial in both the fundamental study of 2D physics and their incorporation in optoelectronic and quantum devices. A direct method to probe charge carrier dynamics with momentum resolution is time- and angle-resolved photoemission spectroscopy (TR-ARPES). Such measurements have been challenging, since photoexcited carriers in many 2D monolayers reside at high crystal momenta, requiring probe photon energies in the extreme UV (EUV) regime. These challenges have been recently addressed by development of table-top pulsed EUV sources based on high harmonic generation, and the successful integration into a TR-ARPES and/or time-resolved momentum microscope. Such experiments will allow direct imaging of photoelectrons with superior time, energy, and crystal momentum resolution, with unique advantage over traditional optical measurements. Recently, TR-ARPES experiments of 2D transition metal dichalcogenide (TMDC) monolayers and bilayers have created unprecedented opportunities to reveal many intrinsic dynamics of 2D materials, such as bandgap renormalization, charge carrier scattering, relaxation, and wavefunction localization in moiré patterns. This perspective aims to give a short review of recent discoveries and discuss the challenges and opportunities of such techniques in the future.

## Abstract 2

Many unique properties in two-dimensional (2D) materials and their heterostructures rely on charge excitation, scattering, transfer and relaxation dynamics across different points in the momentum space.

## Graphical abstract

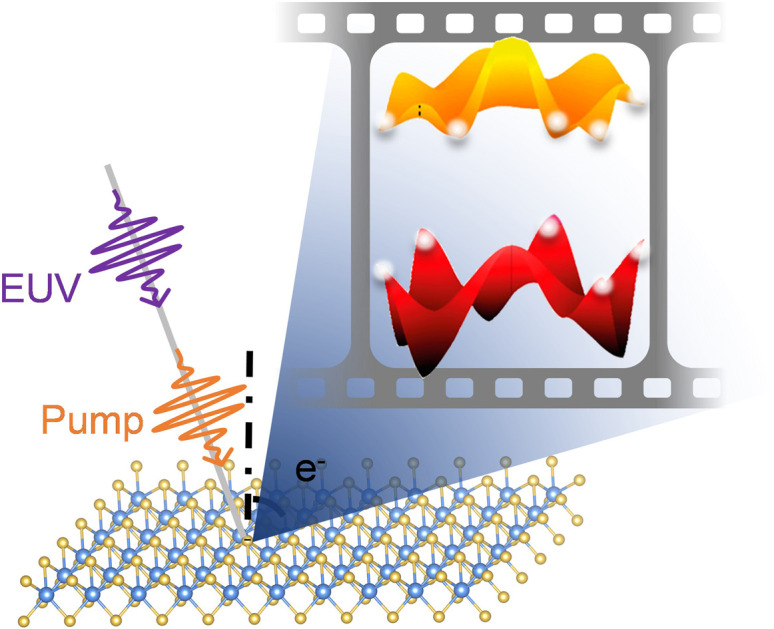

## 1. Introduction

During the past two decades, two-dimensional (2D) atomically thin monolayers have demonstrated a variety of distinct mechanical, optical, and electrical properties that differ from their bulk counterparts, which have stimulated tremendous advances in multiple fundamental research topics.^1,2^ For example, transition metal dichalcogenides (TMDCs), such as MoS_2_, WS_2_, MoSe_2_ or WSe_2_, are monolayer semiconductors with direct bandgap and strong optical absorption in the visible to near IR region. The atomically thin 2D geometry leads to enhanced excitonic effects and reduced charge carrier screening.^3^ In TMDC monolayers, spin–orbit spitting and *C*_3_ symmetry result in selective optical excitations at different inequivalent but energetically degenerate valence band maxima and/or conduction band minima, *i.e.*, valleys.^4,5^ Distribution of carriers, such as electrons and holes, at different valleys can carry information with their corresponding spin and valley indexes. This new route of encoding information, *i.e.*, valleytronics, will offer an innovative approach for information storage and processing, multifunctional quantum devices, and quantum computation.^6,7^ In addition, bilayer structures constructed by stacking two monolayers exhibited emergent phenomena beyond those found in single monolayers. For example, TMDC heterostructures form interlayer excitons with long-lived spin and valley polarizations.^8–10^ The interlayer excitons can be trapped in moiré superlattices,^11^ akin to a matrix of interacting quantum dots.^12–15^ Twisted bilayer graphene at magic angle induces unconventional superconductivity and has promoted the study of new topological properties and correlated electronic behaviors.^16–20^ The unique properties of 2D monolayers and their heterostructures have launched lots of exciting opportunities for future electronic, optoelectronic, and quantum information device applications.^9,17,18,21,22^

The behaviors of electron wave functions in 2D materials in the momentum space dictate the ability of charge carriers to interact with light and phonons through energy and momentum conservation.^23^ Energy- and momentum-resolved dynamics of charge carriers in 2D materials is crucial in understanding many properties such as valley polarization,^6^ interlayer charge transfer,^24^ and exciton localization in moiré superlattices.^25^ Such information has been initially investigated through static optical spectroscopic experiments including optical absorption,^26^ reflection,^27^ and photoluminescence,^28^ as well as time-resolved pump-probe spectroscopy such as transient absorption/reflection^29^ and 2D electronic spectroscopy.^30^ However, most optical spectroscopy experiments measure the bright, momentum-direct optical transitions and are largely restricted to the excitonic phase space regions where interconversion with photons is directly allowed. For example, bright direct bandgap transitions of monolayer TMDCs mostly occur between the band edges at the *K* point. The spectroscopic probing of interlayer excitons of heterobilayers is only sensitive to transitions within the light cone.^31^ However, upon photoexcitation, the charge carrier wavefunctions can relocate to other momenta and have a broader distribution in momentum space. Such scattering processes may play prominent roles in dynamics of the interlayer charge transfer, the formation of indirect or momentum-forbidden dark excitons, and the relaxation of valley polarization.^24,32^ Unfortunately, processes involving the electrons and holes at different momenta often lack bright optical transition to be probed with optical spectroscopy. Despite the versatility of optical spectroscopy measurements, full momentum-resolved dynamics of 2D materials has been unachievable in conventional direct optical detection.

Time- and angle-resolved photoemission spectroscopy (TR-ARPES) provides a direct and superior way to probe band structures and charge carrier dynamics in the momentum space, and reveals the critical information that has been undetectable in optical spectroscopy. Over the past decade, a successful combination of table-top femtosecond EUV generation and TR-APRES has enabled lots of recent studies to unveil the electron dynamics in a variety of quantum materials.^33,34^ For 2D materials, advances in space-, time-, and angle-resolved photoemission spectroscopy techniques allowed for detection of full momentum space information with high spatial and temporal resolutions. They have created unprecedented opportunities in the study of static and dynamic properties in 2D materials. The earliest TR-ARPES experiments on 2D materials were performed on graphene. They revealed ultrafast non-equilibrium response of electrons under different excitation energies near the *K* point of the Brillouin zone and provided crucial information on the electron phonon coupling and the thermalization of electron gas at the Dirac cone.^35–38^ Following the success of graphene, TR-ARPES has recently successfully revealed many of the fundamental underlying quantum behaviors and mechanisms in TMDC monolayers and bilayers with high temporal, spatial, and momentum resolutions. These efforts directly access the extensive dynamics, such as the size and valley configuration of intralayer excitons and interlayer excitons, dark exciton formation, the intervalley scattering dynamics, and how the excitons are influenced by the moiré potential.

One of the major challenges in current research of 2D materials is the availability and quality of samples.^39^ To obtain clear and ordered band dispersion, the TR-ARPES samples usually need to be uniformly crystalline beyond the area of detection, typically on hundreds of micrometers (μm) to millimeters (mm) scale. However, many 2D material samples, including monolayers produced from scotch tape exfoliations, have lateral dimensions of tens of μm. This mismatch has been recently resolved from two general directions: (a) development of time-resolved momentum microscopy (TR-MM), which is based on the electron optics of electron microscopy, to spatially resolve μm-sized regions on small 2D flakes and (b) production of high-quality single crystal monolayers and heterostructures on a large uniform area of mm to cm scale.

Here, we aim to summarize a few recent research developments on dynamic TR-ARPES measurements of 2D materials, focusing on TMDC monolayers and their 2D heterostructures, along with a discussion about future instrument upgrades for further spatial-, spin-, and time-resolved acquisitions. Combined with the experimental advances to prepare large-area, higher-quality monolayers, the momentum- and energy-resolved measurements will reveal new information on exciton creation, relaxation, and charge carrier scattering and localization dynamics beyond those observed in optical spectroscopy experiments.

## 2. Technical overview

### 2.1. A brief overview of TR-ARPES techniques

**Fig. 1:**
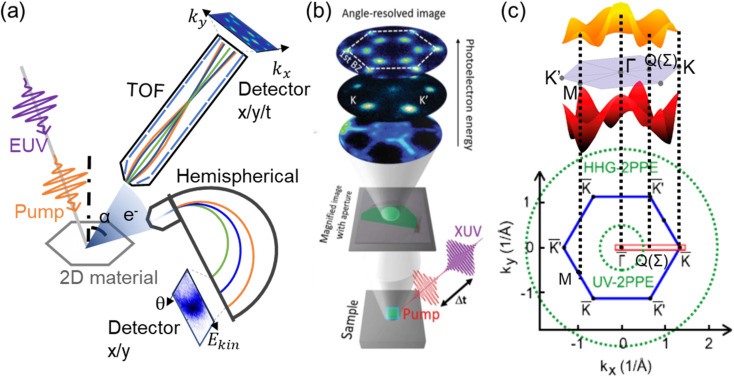
(a) Layout of a typical setup for TR-ARPES of 2D materials, including schematics of both hemispherical (bottom) and time-of-flight (top) electron analyzers. Note that in most experiments, only one electron analyzer is applied. (b) Schematic of a TR-MM experimental setup with magnified real space image plane and reciprocal space plane with angle-resolved images of photoelectrons at different energies, shown for monolayer WSe_2_.^45^ (c) Top: illustration of typical band topologies of 2D TMDCs (shown as 2H bilayer TMDs)^46^ and bottom: Brillouin zone of a TMDC crystal (MoS_2_) with high symmetry points. Dashed green lines represent the observable parallel momentum range using typical tabletop UV probes (UV-2PPE) and EUV probe pulses from HHG (HHG-2PPE).^47^ Copyright (2020) American Association for the Advancement of Science. Copyright (2017) American Chemical Society. Copyright (2016) American Institute of Physics.

ARPES is an effective technique to measure electronic band structures. Static ARPES has played a central role in the recent discovery, characterization, and understanding of quantum materials, which have been extensively reviewed.^34,40–44^ Adding an optical pump prior to the photoionization probe allows for time-resolved measurements, offering unique possibilities to directly measure charge carrier dynamics with time, energy, and momentum information. As shown in Fig. 1(a), a photoemission experiment measures electrons emitted from a sample upon photoionization. The intensities of photoelectrons are resolved in a range of kinetic energy *E*_k_ and angle *α* with respect to the surface normal. The parallel momentum vector *k*_∥_ of photoelectrons from 2D materials can be determined as

1 *k*_∥_ = (2*mE*_k_)^1/2^ sin *α*

The kinetic energy *E*_k_ of photoelectrons reflects the binding energy of the electronic bands where electrons arise from, while the parallel momentum *k*_∥_ of photoelectrons is equal to the crystal momentum of electronic bands in a 2D material. By combining photoemission signals at different kinetic energies and momenta/angles, a slice of the static band structure in *k* space is obtained.

A typical detector used to achieve this goal is a semispherical analyzer shown in the bottom part of Fig. 1(a). After the electrons enter a narrow slit at the entrance, their trajectories are bent by an electric field. The final radii of the electron trajectories depend on their initial kinetic energy. Traditional hemispherical analyzers collect electrons within a small angle range (therefore a small *k*_∥_ range) in a single acquisition, and the scanning of a broader *k* space is traditionally carried out by rotating the sample. Another class of electron analyzers are time-of-flight (TOF) analyzers, shown in the upper part of Fig. 1(a). Electrons traveling at different kinetic energies are separated in time inside a drift tube, and spatially resolved at different angles.^48^ Recent developments of analyzer systems allow for a large solid angle acceptance, which records over a broad (*k*_∥_, *E*_k_) parameter space without sample rotation or angular scanning. Keeping sample position stable is particularly important for spatially resolved ARPES measurements.

Typical scotch tape exfoliated flakes and heterostructures often have lateral sizes of <100 μm. The samples are usually composed of several crystallographic domains with different thicknesses and twist angles, which is not suitable in the traditional TR-ARPES technique with large sampling areas. Time-resolved momentum microscopy is a TR-ARPES technique to solve this problem with μm scale spatial resolution. In a momentum microscope setup, photoelectrons are usually accelerated by an electrostatic electron objective lens, and further transferred, magnified, and projected on to a CCD camera, allowing spectro-microscopic imaging for small samples.^49–51^ Photoelectron microscopy imaging in full-field view can provide spatial resolutions down to the ∼10 nm scale, whereas for clear momentum-space imaging of band structures, the aperture typically samples μm sized areas. A brief schematic is shown in Fig. 1(b). TR-MM has succeeded in resolving the dynamics among different local structural disorders, such as the edges and interior regions of 2D flakes.^52,53^ It is a suitable technique for small exfoliated 2D flakes and their heterostructures.

At the current state of development, the detection capabilities of momentum microscopes and hemispherical analyzers are complimentary to each other, as demonstrated in a recent study with bulk WSe_2_.^54^ A time-of-flight momentum microscope permits efficient mapping of the full band structure and provides an overview of all relevant carrier relaxation pathways within the entire Brillouin zone. On the other hand, a hemispherical analyzer is less restrictive to space charge or detector saturation. A hemispherical analyzer is better at analyzing dynamics within specific energy momentum regions. It has high momentum resolution, rapid data acquisition time, and smaller dataset sizes.

### 2.2. Development of light sources

Many important physical phenomena of 2D or quantum materials occur at momenta far away from the Brillouin zone center *Γ* point, such as the Dirac cone of graphene,^1^ band edges of transition metal dichalcogenides,^3^ and band inversion of many topological materials.^55,56^ According to eqn (1), obtaining information at high momentum *k*_∥_ requires photoelectrons emitted with high kinetic energies, which must be excited by light with large enough photon energy. For example, conduction band and valence band edges of TMDCs are located at the *K* points at the Brillouin zone boundaries. As shown in Fig. 1(c), observation of carrier dynamics at these high momenta often requires probe photon energies >∼15 eV, at the extreme ultraviolet (EUV) region. A traditional way to generate UV probe light for two photon photoemission (2PPE) is frequency doubling/tripling of the tabletop lasers using nonlinear crystals.^57,58^ However, a similar process cannot be used to generate EUV emission, due to significant absorption of common nonlinear crystals at EUV energies beyond their optical bandgaps. Generation of ultrafast EUV probe pulses with sufficient fluence and short pulse duration is a major task in TR-ARPES experimental design. Common sources of EUV radiation include synchrotron/free electron laser facilities^50^ and tabletop high harmonic generation (HHG). EUV from synchrotron radiation has been often applied for resolving static band structures with high energy resolution, while the ultrafast EUV pulses generated from HHG are broadly used in pump-probe TR-ARPES with superior femtosecond time resolution.

**Fig. 2:**
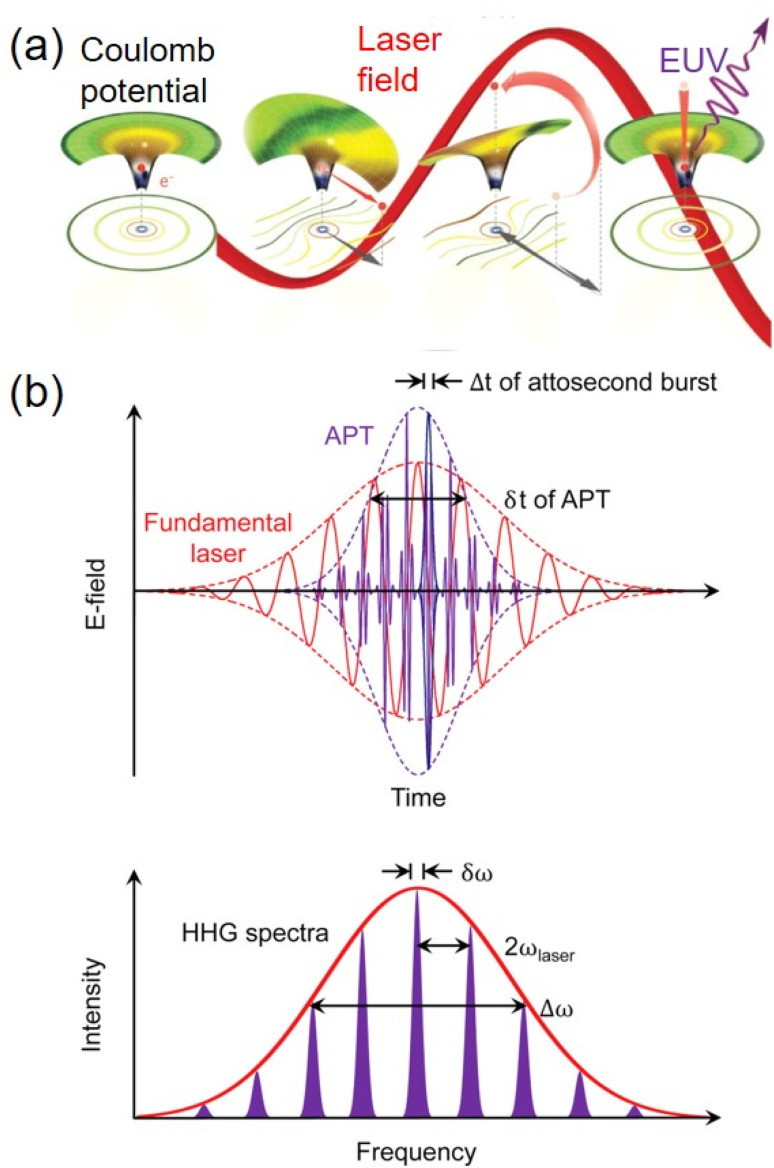
(a) Classical schematic of a three-step model for high harmonic generation. The electric field of an intense laser extracts an electron from an atom through tunnel ionization. The laser field then accelerates the electron, with a small fraction of the electron returning back to the atom, releasing high-energy photons as an attosecond EUV burst.^59^ (b) Time-domain and frequency-domain relationship between the attosecond pulse train “APT” and the HHG frequency comb.^60^ Copyright (2010) Springer Nature.

HHG light sources to generate femtosecond EUV radiation have developed very rapidly in the past few decades, which were reviewed extensively.^59,61,62^ A brief 3-step schematic for the HHG process to generate EUV pulses is shown in Fig. 2.^59^ A laser beam with high intensity is focused into a noble gas. The resulting ponderomotive energy of electrons from the intense laser is much larger than the ionization potential of the gas atoms. As a result, ionization can occur by the tunneling mechanism, when the Coulomb barrier is significantly shaped by the laser electric field. A small proportion of the electrons driven by the oscillatory electric field will return to the atomic nucleus periodically. When they travel close to the nucleus, sudden acceleration of electrons by the intense nucleus attraction generates attosecond bursts of light.^59,61^ Half of the electrons will be ejected near the positive maximum of the electric field while the other half will be ejected near the negative maximum. As a result, the emitted sequence of pulses occurs each half cycle of the driving laser field. Fourier transform of the periodic emission in time creates a spectrum of odd harmonics spaced by two times the fundamental driving laser frequency. The high-harmonic generation is not a perturbative (*i.e.* not a *χ*^q^) process, and the output can be distributed over a wide range of harmonics.

Because certain energy resolutions of TR-ARPES often require a quasi-monochromatic light source, such requirement imposes a trade-off among HHG photon energy, flux, and time resolution. To obtain the desired EUV photon energy, spectral width, and pulse duration, the fundamental driving laser pulse wavelength, intensity, and duration are carefully tuned. A short fundamental pump wavelength yields lower EUV photon energies from HHG, however with higher conversion efficiency.^60,63^ Compared with IR or near-IR fundamental pump frequencies, the EUV fluence can be improved by orders of magnitude through pumping HHG with shorter wavelengths in the visible or UV region. For example, HHG of frequency-doubled Ti:sapphire lasers at 400 nm creates a bright 7th harmonic in Kr gas.^60^ This scheme generates 22 eV EUV radiation, which has been broadly used for many TR-ARPES experiments.^64,65^ Experimentally, the noble gas in TR-ARPES setups can be introduced with small gas-filled cells,^64–66^ gas-filled fibers,^67,68^ gas jet or nozzles.^69–72^ To optimize the EUV fluence, the gas filled capillary waveguide is designed to balance the neutral gas and free electron plasma dispersion and improve phase matching,^66^ and the gas-jet or nozzles is coupled with tight focusing geometry.^62^ The energy resolution of ARPES can be enhanced by adding a EUV monochromator. Using gratings or selectable multilayer bandpass mirrors and thin film filters, recent TR-ARPES studies have achieved a high energy resolution down to 9 meV.^68,73^

To ensure sufficient EUV output in TR-ARPES experiments, the visible or IR femtosecond pump lasers for high harmonic generation often need to be at intense powers of 100 μJ to several mJ per pulse. This has been initially achieved with Ti:sapphire amplifier systems with repetition frequencies of a few kHz.^74–76^ The high density of generated photoelectrons often exhibits electrostatic repulsion among each other, *i.e.* space charge effect, which is a major limitation in ARPES energy resolution.^77^ It can lead to broadening of the photoemitted electron energy spectrum and blurring of the ARPES image. To solve this problem, high harmonic sources operating at higher repetition rates are often desired. The high repetition rate will provide sufficient cumulated signals at a short acquisition time even with fewer photoelectrons per pulse, and minimize the electron–electron repulsion. Recent development of HHG TR-ARPES setups has improved the repetition rates to hundreds of kHz and up to 88 MHz, using Yb fiber laser or Ti:sapphire long cavity oscillators.^69,72,73,78^ HHG efficiency from the low pulse energy at MHz repetition rate can be enhanced by optical cavities, obtaining both high EUV output fluence and minimal space charge distortions in the TR-ARPES.^69,78^ Recently, high-power ytterbium lasers and amplifier systems with both high photon energy and high repetition rates have become commercially available. They allowed efficient EUV production at the hundreds of kHz repetition rate even without HHG cavity enhancement.^63,71,79^ The power of EUV output is up to milliwatt, which opened up new possibilities for TR-ARPES experiments.^63,80^

### 2.3. Development of 2D material samples

Conventional TR-APRES detection requires crystalline samples with a clean, smooth, and uniform area of millimeters to obtain clear band dispersion. In addition, flat sample surfaces are also favored in momentum microscopes to prevent field emission from the high extractor voltage. Current strategies to produce 2D monolayers for TR-ARPES include (a) bottom-up methods, using molecular precursors to synthesize monolayer 2D crystals^81^ and (b) top-down methods, where a monolayer is exfoliated from a layered bulk crystal.^82^ After many years of development, both methods have been reported to produce single crystal 2D monolayer flakes up to millimeter sizes. The availability of large area, high-quality single crystal flakes will open up new possibilities for implementation in large scale devices and the study of their dynamics with TR-ARPES.

**Fig. 3:**
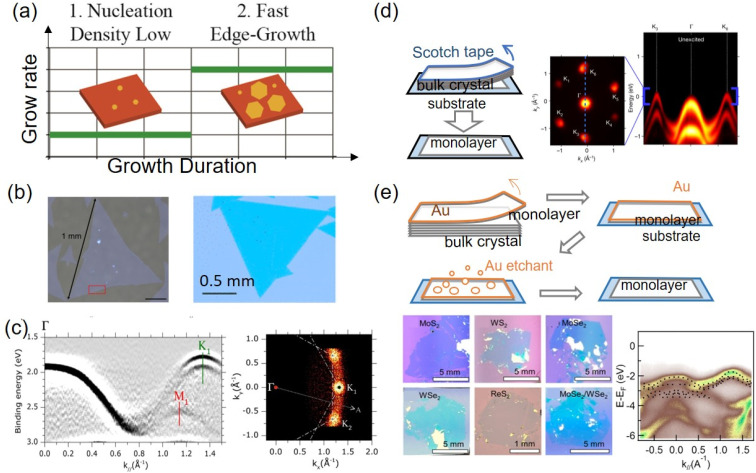
(a) Schematic of a general CVD growth strategy to obtain large single crystal flakes, where low nucleation densities and rapid edge growth rates are favorable, illustrated for CVD graphene.^83^ (b) Examples of large CVD grown TMDC single crystal flakes. Left: MoS_2_;^84^ right: MoSe_2_ (ref. 85) (c) left: ARPES electronic band structure of a CVD-grown continuous MoS_2_ monolayer. Right: constant energy plot taken around the valence band maximum at K.^86^ There are two preferred orientations of the MoS_2_ monolayer grown on sapphire, leading to two set of bands K_1_ and K_2_ overlapping with each other at 30° angle mismatch. (d) Left: schematics of scotch tape exfoliation. Right: constant energy plot and energy–momentum dispersion along the *K*–*Γ* direction of a WSe_2_/MoS_2_ heterobilayer constructed from scotch tape exfoliated monolayers, obtained in a TR-MM setup.^87^ (e) Top: schematics of gold tape exfoliation; bottom: optical images of the obtained monolayer and heterostructure, and ARPES of MoSe_2_/WSe_2_ heterobilayers prepared by gold tape exfoliation.^88^ Copyright (2016) (2017) (2018) American Chemical Society. Copyright (2022) Springer Nature. Copyright (2020) American Association for the Advancement of Science.

The most recognized bottom-up synthesis technique is chemical vapor deposition (CVD), which has unique advantages in high scalability and low cost. A general strategy to grow large flakes is suppressing the nucleation density^89,90^ and enhancing the edge growth speed,^91^ as shown in Fig. 3(a). It yields single crystal graphene flakes with a lateral dimension of mm to cm scale.^83,92^ For monolayer transition metal dichalcogenides, large flakes are obtained by lowering nucleation density through reactive barriers,^86^ a molten glass surface,^85^ or self-capping vapor–liquid–solid reactions.^93^ As such, mm-scale single-crystal TMDC monolayers or full-coverage polycrystalline TMDC films with large domain sizes can be synthesized. Examples of the obtained large TMDC flakes are shown in Fig. 3(b). The large monolayer films have demonstrated a well-resolved band structure in ARPES, as shown in Fig. 3(c).^86^ On the other hand, several recent advances have been made to grow homobilayer crystals and heterostructure bilayers such as TMDC/graphene.^94–96^ Most of the grown heterobilayers are with a 0 or 60° twist angle and have μm or sub-μm-sized domains. They have been applied in the study of ultrafast interlayer hot carrier transfer dynamics with optical spectroscopy and TR-ARPES experiments, and integrated into photodetectors.^97–99^ However, direct growth of twisted bilayers and/or heterobilayers with designed nonzero uniform twist angles over a large area has been particularly challenging and awaits further developments.

The most popular top-down exfoliation strategy is tape exfoliation, which is shown in Fig. 3(d). It has been widely used to produce high-quality monolayers with lateral dimensions up to tens of μm.^2,100^ Compared with CVD-grown flakes, single-crystal flakes from Scotch tape exfoliation are often of higher quality and better surface cleanness, benefiting from the low defect density of bulk crystals grown with the chemical vapor transport or flux growth methods.^101^ In comparison with Scotch tape and Si substrates, metals are found to be more efficient exfoliation media.^102^ The strong metal–monolayer interaction effectively overcomes the interlayer van der Waals attractions.^103^ Metals also induce a lateral strain on the target monolayer to enhance monolayer selectivity.^104^ As shown in Fig. 3(e), metal-assisted exfoliation has obtained monolayers with lateral dimensions of mm to cm.^88,105–108^ The large monolayers can be used to construct bilayer and multilayer stacks, with desired twist angles that are uniform over the mm-sized sample.^88,109^ The obtained single crystal monolayer and heterostructures displayed well-resolved reciprocal-space band structure and crystal structures in photoemission^88^ and electron diffraction^110^ and can be an excellent material platform for TR-ARPES experiments.

In a typical APRES experiment, ejection of photoelectrons leaves a net positive charge on the sample. Therefore, the 2D material sample needs to be electrically grounded to prevent charge accumulation. A number of TR-APRES studies, including a few of the earliest experiments, were conducted with monolayers on a metal substrate such as Au, or a conductive substrate such as HOPG or graphene.^74–76^ The results revealed many key dynamics such as charge carrier extraction from metal contacts. However, monolayers are highly susceptible to environmental screening from a conductive substrate. A metal substrate has a strong n doping effect on the monolayers,^107,111^ which significantly reduces the charge carrier lifetimes, closes the bandgap, and changes the CB/VB alignment in TMDC monolayers.^112,113^ In addition, mismatches of the lattice and stiffness between the metal and monolayer will jointly lead to epitaxial stress and biaxial strain on the monolayers grown on metal.^111,114–116^ A number of recent TR-ARPES experiments are conducted on monolayers or bilayers positioned on a dielectric substrate such as SiO_2_ or hBN.^45,87,109,117,118^ The electrical grounding is achieved either by placing an electrode on the side of the monolayer crystal or by contacting through a very thin hBN layer on a highly doped Si substrate. The dielectric substrates will maintain photoexcited charge carriers at higher densities with longer lifetimes on hundreds of ps timescale.^117^ It allows probing of more pristine properties of monolayers or bilayer 2D materials.

Photoemission spectroscopy is highly sensitive to the surface of the sample. For bottom-up CVD growth, large monolayer TMDC flakes are usually grown on insulating substrates such as sapphire and SiO_2_/Si. On the other hand, most monolayers from common top-down exfoliations are initially prepared on SiO_2_/Si substrates as well, for better visualization and sample screening. These prepared monolayers often need to be transferred onto a new destination substrate, such as metal or thin hBN/Si for ARPES measurements. The transfer or stacking of 2D materials often involves many steps such as polymer coating and/or solvent rinsing. The residual surface contaminations and air adsorption require further treatments, such as annealing in forming gas and vacuum, to obtain a clean surface before a TR-ARPES measurement can be conducted.

## 3. TMDC monolayer dynamics

The poorly screened Coulomb potential in 2D monolayers leads to exceptionally large exciton binding energies (*E*_b_). In the presence of charge carriers, the many-body Coulomb interactions and dielectric screening will reduce exciton binding energies and shrink the bandgaps (*E*_g_); the latter effect is described as bandgap renormalization. The amount of reduction in *E*_g_ and *E*_b_ is almost the same. The optical transition energy measures the difference between them, and their cancellation often leads to little change in the optical transition energies at different carrier densities.^119,120^ An ideal technique to measure quasiparticle energy is TR-ARPES, which enables the precise determination of the CB/VB energies with momentum resolution. Multiple research efforts have been carried out on this aspect, revealing monolayer bandgaps under different local or transient charge carrier density and/or dielectric screening conditions.

A typical material platform to study the reduction of bandgaps of monolayers is on metal substrates, which provides both intense dielectric screening and efficient charge carrier extraction. An early TR-ARPES study of MoS_2_ monolayers epitaxially grown on Au (111) directly observed momentum-space ultrafast free carrier dynamics.^74^ The result determined a direct quasiparticle bandgap of 1.95 eV, substantially smaller than the estimated value of 2.8 eV for free-standing MoS_2_. The dynamic measurement reveals an ultrafast (50 fs) transfer of excited free carriers from monolayer MoS_2_ to the Au substrate. The hot electron transfer dynamics between monolayer MoS_2_ and Au is later investigated using TR-MM with a 2.4 eV pump and 3.61 eV probe, with an advantage to spatially distinguish photoemissions from different areas in micrometer-sized samples.^121^ The electron transfer at the MoS_2_/Au interface was found to occur via incoherent hopping. Its dependence on local MoS_2_/Au distances is confirmed by AFM characterization of the same area. An in-depth TR-ARPES study on monolayers MoS_2_ and WS_2_ grown on Au(111), Ag(111) and graphene/SiC further revealed the transient electronic structure and ultrafast carrier dynamics.^122^ The monolayer on Au and Ag exhibited a similar reduced quasiparticle bandgap and efficient removal of photoinduced carriers from monolayer into the metal. In comparison, the monolayer bandgap is much larger (∼2.5 eV) on graphene, indicating that graphene has less of dielectric screening effect than metals. The initial decay time constant of the CB electrons for TMDC/graphene is on the order of 170 fs, slower than TMDC on metal substrates, and was attributed to Auger recombination and in gap defect states.

**Fig. 4:**
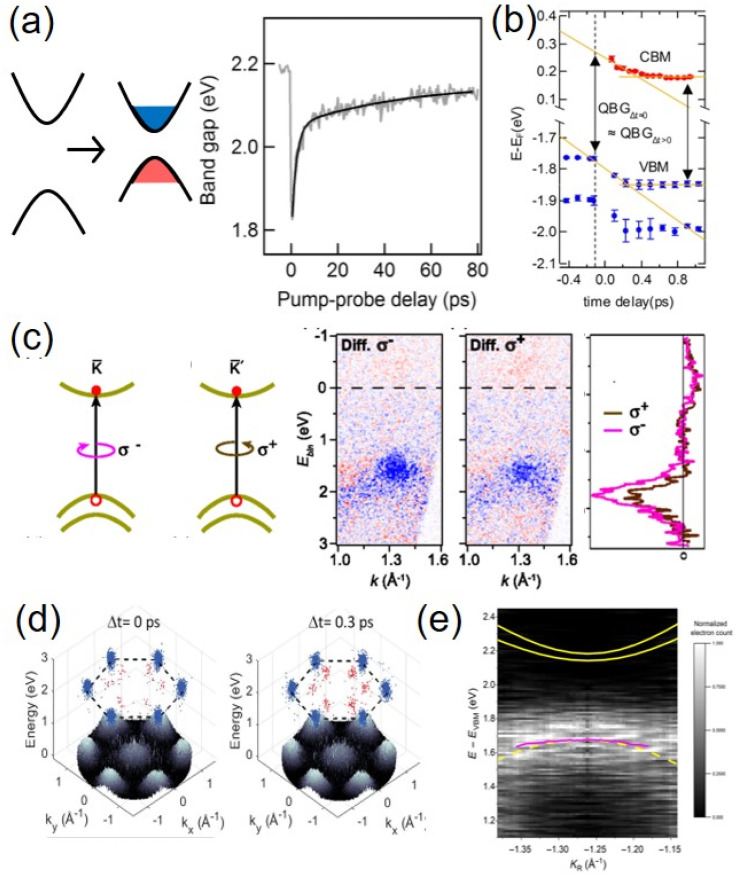
Dynamics of monolayers revealed by TR-ARPES. (a) Band renormalization of monolayer MoS_2_ at different pump probe delay times, at high excitation density (200 μJ cm^−2^).^117^ (b) CBM and VBM energies of MoS_2_ at different pump probe delay times under much lower excitation density (a few tens of μJ cm^−2^).^123^ (c) Valley polarization of the valence band holes in the WS_2_ monolayer, shown as difference signals at the K valley for optical pumping with *σ*^−^ and *σ*^+^ circular polarization in the middle, and difference in the corresponding energy distribution curves on the right. Red: increase; blue: decrease of signals.^76^ (d) Full momentum map from TR-MM illustrating exciton dynamics of monolayer WSe_2_ after resonant excitation. The top of the valence bands is displayed in grayscale; emission from the K-valley (Q-valley) excitons is plotted in blue (red) dots.^45^ (e) Experimental energy–momentum distribution of the electrons photoemitted from an exciton, along a 1D cut in *k*-space centered at the K-valley. Notably, the CB exciton signal shows a negative dispersion resembling the VB. The yellow solid curves are the theoretical spin–split CB free electrons.^118^ Copyright (2017) (2019) American Physical Society. Copyright (2021) American Chemical Society. Copyright (2020) American Association for the Advancement of Science.

The charge carriers generated from photoexcitation also induce strong screening effects, leading to the renormalization of quasiparticle bandgaps in the 2D material. Photoexcitation with pump fluences above the Mott threshold creates electron–hole gas inside a monolayer. A TR-ARPES experiment was conducted on the MoS_2_ monolayer exfoliated on SiO_2_/Si upon such high excitation density. As shown in Fig. 4(a), it directly reveals a reduction of bandgap up to 400 meV.^117^ The measured quasiparticle bandgap provided an experimental benchmark for strong many-body interactions in the TMDC monolayer, which shows excellent agreement with later computational studies.^124^ Another TR-ARPES study carried out on monolayer MoS_2_ on graphite substrates characterized the intravalley cooling dynamics of CB electrons around the *K* point.^123^ A negligible quasiparticle bandgap renormalization was observed (displayed in Fig. 4(b)). At low excitation density, the dielectric screening effect from HOPG is dominant over the screening from photoexcited free carriers. As opposed to the bandgap reduction from screening of free carriers, an *increase* in the bandgap by 40 meV was recently observed in the monolayer MoS_2_/HOPG system.^125^ The TR-ARPES experiment was carried out at lower excitation densities below the Mott threshold, which created excitons in MoS_2_ instead. This phenomenon was assigned to exciton-driven band renormalizations due to the simultaneous enhancement of band effective mass, which was supported by excitonic many-body theoretical calculations.

A unique property of TMDC monolayers is their valley polarizations. TR-ARPES is an essential tool to understand the allocation and relaxation of optically excited free carriers at different valleys in a momentum-resolved electronic band structure. Among the different TMDC materials, WS_2_ and WSe_2_ exhibit a large spin–orbit splitting of ∼400 meV in their valence band, making them great candidates to demonstrate the spin and valley polarization of charge carriers. An early TR-ARPES study demonstrated valley polarized excited holes in single-layer WS_2_ grown on Ag(111).^76^ Circularly polarized optical excitation created holes preferably within upper spin split branch of a certain valence band valley. The results are shown in Fig. 4(c), demonstrating that the K valley was preferably excited with σ-circular polarization. Compared with the large VB spin orbit splitting, the CB splitting of TMDC monolayers is much less. The CB electrons are therefore subject to a higher degree of valley depolarization and intervalley coupling. Using TR-MM with both linearly and circularly polarized excitation, the intervalley and intravalley coupling dynamics of CB electrons were recently characterized in monolayer WS_2_ exfoliated on hBN.^126^ The result shows that the dominant valley depolarization occurs *via* the intervalley Coulomb exchange mechanism. It also revealed strong mixing between B_1s_ and A_*n*>1_ excitonic states in monolayer WS_2_.

As shown in Fig. 1(c), the *Q* point is another local minimum in the CB of TMDC monolayers in addition to the band edges at the corners. Electrons residing at the *Q* point will give rise to dark K–Q excitons, where direct transition is optically forbidden. Dynamics at multiple valleys across the entire Brillouin zone can be directly probed with a momentum microscope, providing superior spatial, time, energy, and momentum resolution. Using TR-MM with a UV probe pulse of 273 nm, Li *et al.* were able to resolve CB electron cooling dynamics at *Q* points for both supported and suspended monolayer WS_2_ on a time scale of 300 fs.^127^ Madéo *et al.* used TR-MM with an EUV probe to resolve the peak distributions for both bright K–K exciton and momentum-forbidden K–Q dark excitons in monolayer WSe_2_ on hBN, with clear resolution in time, momentum and energy, as shown in Fig. 4(d).^45^ With resonant excitation, dark Q valley excitons were formed through scattering from the K valley excitons on an ∼400 fs timescale. With above bandgap excitation, carrier cooling and relaxation in the K or Q valley occurred within 500 fs.

TR-ARPES has long been used to characterize the free carrier dynamics and band structures as signatures of single-particle states. Excitons, which are localized electron–hole bound states, have only recently been mapped out directly in the momentum space through experimental TR-ARPES of monolayer TMDCs. Compared to free electrons, removal of electrons from bound electron–hole pairs in excitons leaves behind the hole with energy conservation. As a result, the angle-resolved photoemission of excitonic electrons will reflect the photohole quasiparticle band structure, creating an inverted energy–momentum dispersion relationship. The feature will be located below the conduction band minimum, displaced by the exciton binding energy.^128,129^ This has been difficult to resolve in conventional bulk semiconductors where the exciton binding energy is small (10 meV).^130^ However, TMDC monolayers host excitons with significantly larger binding energies in hundreds of meV, providing new opportunities to observe excitonic signatures in photoemission measurements. Man *et al.* recently measured the intrinsic excitonic wavefunction of monolayer WSe_2_ using TR-MM, and achieved high resolution image of the excitonic wavefunction in the momentum space, as shown in Fig. 4(e).^118^ The result is directly reflective of real space electron distribution around the hole of excitons in 2D materials.

## 4. Bilayer dynamics

### 4.1. Homobilayers

**Fig. 5:**
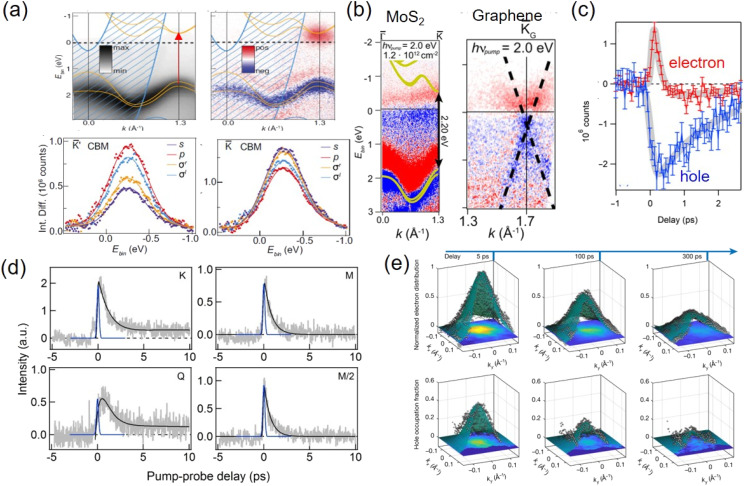
Dynamics of bilayers revealed by TR-ARPES. (a) Top: ARPES of homobilayer MoS_2_ and the intensity changes upon photoexcitation. Bottom: intensity difference of the CB minimum electrons at K and -K valleys, pumped with different polarizations.^132^ (b) Intensity difference APPES spectra along the *Γ*–*K* direction of the MoS_2_ and graphene bands in a MoS_2_/graphene heterostructure, showing renormalized gap at MoS_2_. Red: increase; blue: decrease.^75^ (c) Pump-probe signal of the Π-band of graphene above (red) and below (blue) the equilibrium chemical potential, representing electrons and holes in the graphene layer of a WS_2_/graphene heterostructure.^133^ (d) CB electron dynamics of a WS_2_/MoS_2_ heterobilayer at *K*, *Q*, *M*/2, and *M* points as a function of pump probe delay.^109^ (e) Distributions of electrons and holes of interlayer excitons in a WSe_2_/MoS_2_ heterostructure around the K valley at different delay times.^87^ The momentum space distributions of the excitons are reflective of their confinement in the real space moiré superlattice. Copyright (2019) (2020) American Physical Society. Copyright (2016) American Chemical Society. Copyright (2022) Springer Nature.

Compared with monolayers, bilayers constructed from two TMDC monolayers often require higher complexities in sample fabrication and have been so far less explored in TR-ARPES. TMDC homobilayers often have different static band structures than monolayers. The interlayer band hybridization will shift the alignment of the local VBM or CBM from the *K* point of monolayers to the other momenta such as *Γ* or *Q* points, leading to reduction of photoluminescence intensity.^28,131^ A TR-ARPES study of bilayer MoS_2_ and WS_2_ grown on Au(111), Ag(111) and graphene/SiC observed a redistribution of excited holes in the VB, which were affected by their interactions with bulk metallic states.^122^ Interband transitions of 2D materials can be induced using ultrafast laser pulse with different polarizations, including s, p, left and right-hand circular polarization. As shown in Fig. 5(a), the photoemission selection rules of these different polarizations lead to different CB intensities in TR-ARPES, which were reflective of the symmetry and orbital character of the electronic wave functions.^132^ In the bilayer MoS_2_ grown on Ag(111), the conduction band was found to have a strong linear dichroism effect of up to 42.4%. The strong anisotropic momentum dependence of the optical excitation is supported by theoretical calculations, and their occurrence is due to intralayer single-particle hopping.

### 4.2. Heterobilayers

TMDC/graphene is a popular 2D bilayer heterostructure that has been heavily investigated with TR-ARPES. Compared with metal substrates such as Au or Ag, graphene offers a much weaker dielectric screening on TMDC monolayers and serves efficient grounding. Band structures of graphene can also hybridize with TMDCs, leading to avoided crossings between the valence bands of graphene and monolayer TMDC.^134^ However, the hybridization occurs at crossings away from the band edges, which allows for clear distinction of the individual band structures and dynamics of TMDCs and graphene at different momenta. Such structures became excellent platforms for studying both the dynamics of TMDC monolayer and TMDC/graphene heterobilayer systems. Ulstrup *et al.* used TR-ARPES to record the transient band structure evolution of a MoS_2_/graphene heterostructure epitaxially grown on SiC.^75^ The result is shown in Fig. 5(b), which revealed renormalization of the MoS_2_ quasiparticle bandgap up to ∼400 meV from photoexcited charge carriers. Upon photoexcitation, graphene also exhibited a direct transition across the Dirac cone; however, its band energy was not shifted. In a TMDC/graphene heterobilayer, the photoexcited electrons and holes will experience different scattering phase space in the relative band alignments of both layers, leading to different interlayer charge transfer rates and relaxation pathways. The photogenerated electrons and holes on an epitaxial WS_2_/graphene heterostructure were resolved in TR-APRES.^133^ Part of the results are shown in Fig. 5(c). During a short time after the resonant photoexcitation of WS_2_, holes were found to transfer from the WS_2_ layer into the graphene layer, while electrons remained in the WS_2_ layer for a lifetime of ∼1 ps. As such, the heterobilayers formed a new charge-separated transient state in the early times after photoexcitation.

TMDC/TMDC van der Waals heterostructures of two different monolayers is another popular 2D material platform. TMDC heterobilayers with type II band alignment can form long-lived interlayer excitons with electron hole pairs, which are separated both in real space across two monolayers and in momentum space at certain twist angles. Upon photoexcitation, intralayer excitons are created at the *K* point. The electrons and holes can undergo ultrafast scattering into the other momenta, such as the *Q* (or *Σ*) point for electrons and *Γ* point for holes. The orbitals at *K* point are highly layer-localized; however, the orbitals at the other momenta are more hybridized.^24,32^ Such a process will facilitate the interlayer charge transfer. Scattering of the charge carriers back to the *K* point results in formation of interlayer excitons on an ultrafast time scale. In contrast to optical measurement that is limited to transitions at specific light cones of the momentum space, TR-ARPES can provide a full momentum-resolved picture of the charge carrier dynamics in heterostructures. A TR-ARPES study of a heterobilayer WS_2_/MoS_2_ exfoliated on SiO_2_/Si revealed momentum-resolved electron dynamics among different points in the CB.^109^ As shown in Fig. 5(d), during a short time (∼70 fs) following photoexcitation at the K valleys, the electrons are detected in multiple momenta in the Brillouin zone including the *M*/2, *M*, and *Q* points. After longer times of ∼400 fs, the CB electrons relaxed to the lower energy *K* and *Q* points, followed by a dynamic equilibration between them. A recent TR-MM experiment on the WSe_2_/MoS_2_ heterobilayer revealed efficient electron scattering from *K* to *Q* points within 50 fs, and form interlayer excitons on a similar timescale.^135^ These results provide direct evidence of the charge scattering mechanism during the formation and relaxation of interlayer excitons, with clear resolution of charge carrier distributions at different momenta.

When two monolayer lattices in bilayer structures are overlaid with a small twist angle *θ*, they form moiré superlattices with long periodicities. The local variation of atomic stacking in the superlattice will lead to the periodic modulation of the interaction potential (moiré potential). The photoexcited electrons and holes will be trapped in the periodic potential and form moiré excitons.^13–15,136^ The periodic real space superlattice creates a mini Brillouin zone in momentum space from the shrinkage of reciprocal unit cells.^32^ Using TR-MM, Karni *et al.* directly probed clear momentum- and energy-resolved images of electrons and holes in moiré excitons of a WSe_2_/MoS_2_ heterostructure.^87^ The corresponding dynamics is shown in Fig. 5(e). After resonant photoexcitation of WSe_2_, interlayer excitons are created, cooled, and confined in the moiré superlattice. The momentum space distribution of excitons is directly reflective of their real space localization. Compared with the moiré cell length of 6.1 nm, the interlayer excitons have a diameter of 5.2 nm and are localized to a region of only 1.8 nm diameter within the moiré cell. The energy–momentum fingerprints of the moiré interlayer excitons within the mini Brillouin zone have also been recently captured, in a TR-MM experiment of a twisted WSe_2_/MoS_2_ heterostructure.^135^ The results revealed interlayer excitons with a Bohr radius of ∼1 nm, trapped in a 2 nm moiré cell. It provided a direct experimental measurement on how the exciton wavefunction can be modulated within the moiré potential.

## 5. Future outlook

Ultrafast TR-ARPES provides multidimensional detection with direct time, energy, and momentum resolution. Understanding the momentum-space transient behavior of the electronic structure and ultrafast carrier dynamics is crucial to interpret many key properties of 2D materials, such as spin and valley polarization, intervalley scattering dynamics, formation, and relaxation of momentum-indirect dark excitons and moiré excitons. New scientific problems emerging in 2D materials will demand enhanced capabilities to resolve complex and correlated interactions between charge carriers, spin, orbitals, and the crystal lattice, demanding further developments in both ultrafast laser technology and TR-ARPES instrumentation.

Further multidimensional detection of 2D material dynamics will involve both momentum and real space mapping and dynamic wavefunction reconstruction. It will capture important features of 2D materials such as the Fermi surface, Fermi velocity, band dispersion, dichroism, orbital, and spin. In particular, applying spin filter crystals to electron analyzers will enable direct extraction of the spin-dependent spectral density function of 2D materials in energy–momentum space.^137^ The spin-resolved information of the charge carriers will be crucial for understanding the spin polarizations/valley polarizations and corresponding dynamics, which are important for future development of valleytronic devices. Moreover, TR-MM has the potential to access the spectral function of *in operando* devices with superior real space resolution.^42^ As such, it will help obtain detailed multidimensional phase space information of 2D materials in an operating optoelectronic device, including the local band structure, charge carrier dynamics, and many-body interactions.

With the continued progress of TR-ARPES techniques and their applications to 2D materials, the current capabilities in the spatial, temporal, and momentum space resolution will be further pushed toward new limits.^138^ Today, hemispherical analyzers have reached excellent performance with high energy resolution (down to <1 meV)^139,140^ and high momentum resolution (down to ∼0.003 Å^−1^).^141^ However, energy resolutions in currently reported TR-ARPES measurements of 2D materials are only down to the order of ∼10 meV. The energy resolution is largely restricted by (a) the energy bandwidth of the femtosecond EUV probe laser; (b) the inhomogeneity of materials that are susceptible to local dielectric disorder; and (c) space charging from high density photoelectrons at a certain time and space. To achieve the full potential of TR-ARPES, many parameters in the instrumental design, such as the number of electrons per pulse, kinetic energy, the EUV pulse duration, flux, and photon energy, will be optimized.

Although multiphoton ionization of the conventional UV laser has proved a possibility to probe momentum up to 0.6 Å^−1^,^142^ the EUV sources including free-electron lasers and laboratory-based high-harmonic-generation sources remain as the most favorable way to provide fs pulses for full Brillouin zone mapping in TR-APRES experiments, especially for 2D materials. It will demand EUV photon sources with enhanced stability and fluence at high repetition rates. As repetition rates of lasers are approaching MHz rep rates or higher, the energy broadening due to space charging will be eliminated.^69^ To follow the increasing rep rate to higher MHz scale, faster electronic responses in TR-MM may be developed at the same time. Further development of EUV laser sources will provide optimal repetition rate, peak power, pulse duration and energy bandwidth for each specific TR-ARPES application.

To obtain well-resolved band structures, an ideal 2D monolayer sample for TR-APRES will need to be clean and uniform over a large area in both crystal alignment and dielectric environment. Further improvement in the bottom-up and top-down synthesis techniques will be desired. CVD samples are usually grown with high defect densities and epitaxial strain which may require relaxation.^143^ On the other hand, exfoliated monolayers and heterobilayers may have bubbles and local strain induced during transfer and stacking, leading to local disorders.^144,145^ Such inhomogeneities will significantly impact energy and momentum resolution in ARPES spectra. Future development in material preparation techniques may need to achieve production of large area, uniform, flat, and strain-free monolayers and bilayers with low defect densities. Once bilayers with uniform moiré patterns are obtained over a large area, dynamic trapping, and relaxation of moiré excitons can be more clearly resolved at the backfolded bands of the moiré mini Brillouin zone. So far, the majority of the reported TR-ARPES experiments are carried out on graphene and TMDC monolayers and bilayers. With the maturity of efficient large area, high-quality monolayer preparation techniques, a broader range of 2D quantum materials, such as 2D magnetic materials, will be further explored in the near future.

How to directly integrate 2D material preparation into the same ultra-high vacuum system as TR-ARPES is the next major challenge to overcome. Monolayers prepared *ex situ* always require further surface treatments for high-quality ARPES data. The surface treatments, such as rinsing and annealing, may impose risks such as degradation of monolayer sample quality and/or change of small angle twisted bilayer structures. Ideally, the monolayer or bilayer samples could be prepared *in situ* and measured without further cleaning or exposure to the atmosphere. This is fundamentally important for the more complex air-sensitive 2D materials. In this regard, bottom-up synthesis, such as chemical vapor deposition or physical vapor deposition, may have great potential to integrate into the same vacuum system as TR-ARPES. *In situ* material fabrication in combination with tabletop photoemission characterization will be an important future activity to greatly expand the quality and library of TR-ARPES measurements on 2D systems.

## Author contributions

F. L. wrote the manuscript.

## Conflicts of interest

There are no conflicts to declare.

## Supplementary Material
